# Cisplatin‐coordinated copolythiophene for synergistic chemotherapy and sonodynamic therapy of tumor

**DOI:** 10.1002/smo.20240003

**Published:** 2024-06-07

**Authors:** Yuanyu Tang, E. Pang, Pan Zhu, Qiuxia Tan, Shaojing Zhao, Benhua Wang, Chaoyi Yao, Xiangzhi Song, Minhuan Lan

**Affiliations:** ^1^ Hunan Provincial Key Laboratory of Micro & Nano Materials Interface Science College of Chemistry and Chemical Engineering Central South University Changsha Hunan China

**Keywords:** chemotherapy, cisplatin, polythiophene, sonodynamic therapy

## Abstract

Sonodynamic therapy (SDT) is a novel cancer treatment type showing the advantages of high tissue penetration ability, non‐invasion, low systemic toxicity, and high selectivity. However, SDT depends on ultrasound (US) irradiation; once US is turned off, the sonosensitizer will stop producing reactive oxygen species (ROS). Moreover, most sonosensitizers generate oxygen‐dependent ROS, that is, singlet oxygen (^1^O_2_), significantly limiting the therapeutic effect of SDT in treating deep and hypoxic tumor. Therefore, combining SDT with other treatment modalities is essential. Here, we designed and synthesized a series of cisplatin‐coordinated copolythiophenes (**CPT‐Pts**), simultaneously generating ^1^O_2_, superoxide anion, and hydroxyl radicals for synergistic chemotherapy and SDT of tumor. The sonodynamic toxicity and cytotoxicity of **CPT‐Pts** were accurately regulated by tuning the monomer ratio of the polythiophene. This copolymerization strategy avoids the side effects originating from the high‐dose chemotherapy drug while making up for limiting SDT relying on ultrasonic activation, effectively inhibiting cancer cells and tumors.

## Graphical abstract

Sonodynamic therapy (SDT) has attracted much attention due to its excellent tissue penetration ability of US, but it is also limited by its dependence on US excitation. This work designs and synthesizes cisplatin‐coordinated copolythiophene (**CPT‐Pts**), which could produce reactive oxygen species under US, and thus kill cancer cells and inhibit tumor growth by the combination of SDT and chemotherapy.

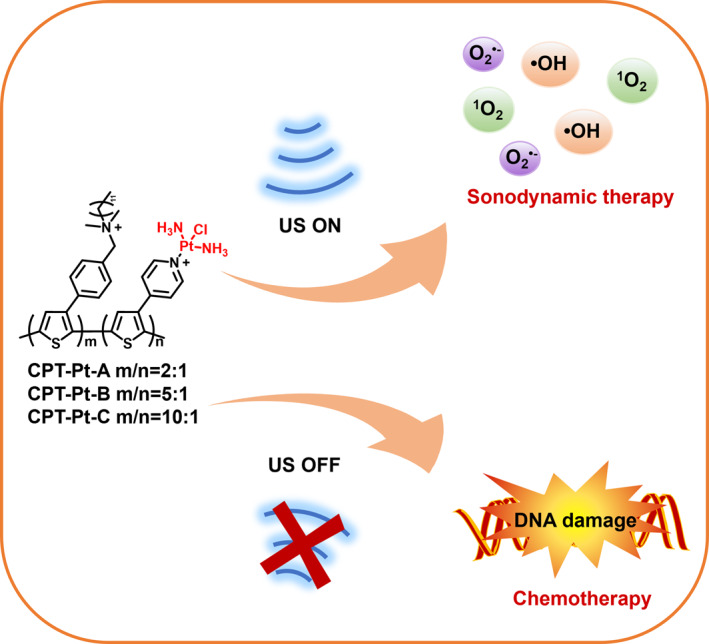

## 1 INTRODUCTION

Surgery, radiotherapy, immunotherapy, and chemotherapy are the clinical cancer treatments. However, the postoperative trauma of surgery, the severe side effects of radiotherapy, and the drug resistance of chemotherapy are challenging to overcome. Moreover, the compactness of tumor tissue makes it difficult for nanomedicines to penetrate solid tumors while delivering drugs to cells far away from blood vessels.^[1]^ Photodynamic therapy (PDT) has the advantages of high selectivity, no drug resistance, minor trauma, and few side effects,[^2^, ^3^, ^4^] which has also been approved for clinical application in treating glioma,^[5]^ vertebral metastasis,^[6]^ periodontitis,^[7]^ etc. The light‐excited photosensitizers react with oxygen to generate reactive oxygen species (ROS), inducing oxidative stress and killing tumor cells.^[8]^ However, due to the absorption and scattering of light in biological tissues, light cannot penetrate deep tissues, limiting the effective treatment of deep tumors using PDT.^[9]^

Sonodynamic therapy (SDT) is a promising emerging treatment with high tissue penetration depth, non‐invasive, and low systemic toxicity.^[10]^ The efficiency and species of ROS generation are crucial factors in the therapeutic effect of SDT. Inorganic sonosensitizers, including titanium‐based nanoparticles, ZnO, and Ag_2_S quantum dots, could generate ROS under ultrasound (US) irradiation.^[11]^ However, their low sonodynamic activity, poor biocompatibility, non‐biodegradability, and potential biosafety issues inhibit their clinical application. In contrast, organic sonosensitizers, such as porphyrins and phthalocyanine, have a precise molecular structure, stable physicochemical properties, high ROS generation efficiency, and biodegradability, which benefit their clinical applications.^[12]^ However, they also suffer from high phototoxicity and poor subcellular organelle targeting capability. Moreover, their poor water solubility also reduces their ability to generate ROS in vivo.

On the one hand, SDT depends on US irradiation. Once the US is turned off, the sonosensitizer will stop producing ROS. Moreover, most sonosensitizers can generate oxygen‐dependent ROS, that is, singlet oxygen (^1^O_2_), significantly limiting the therapeutic effect of SDT in treating deep and hypoxic tumors.[^13^, ^14^] Combining chemotherapy and SDT is an effective strategy to kill deep tumor cells. On the other hand, at the tissue level, the US can remodel the dense and rigid extracellular matrix of heterogeneous tumors and enhance drug carrier penetration.^[15]^ At the cellular level, US‐triggered cavitation develops microbubbles, which vibrate or burst with energy release. This produces unrecoverable sonoporation on the cell membrane and significantly enhances the cell membrane permeability to drug molecules.[^16^, ^17^] Thus, US treatment can promote the internalization of sonosensitizers and chemotherapeutic drugs while improving their therapeutic efficacy. Moreover, excessive oxidative stress destroys the intracellular redox balance and increases the sensitivity of tumor cells to chemotherapeutic drugs.[^18^, ^19^] Therefore, developing a sonosensitizer with good biocompatibility and chemotherapeutic drug payload is of great clinical application for chemotherapy and SDT.

Liu et al. constructed a multi‐functional sonosensitizer (DVDMS‐MN‐LPs) by encapsulating sinoporphyrin sodium chelated with manganese ions inside nanoliposomes.^[20]^ Although the nanoliposomes enhance the biostability of sinoporphyrin sodium, premature release, and insufficient targeting persist. Zhai et al. developed a redox/enzyme/US responsive nanoplatform for antiproliferation and antimetastasis of melanoma to solve the problem.^[21]^ Multifunctional nanoplatforms can enhance the biocompatibility and targeted delivery of sonosensitizers.^[22]^ Cui et al. produced a multifunctional sonosensitizer platform (Hp Ab‐LiP‐ICG) targeting *H. pylori* with photoacoustic imaging and SDT capabilities by loading indocyanine green (ICG) inside monoclonal antibody‐conjugated liposomes.^[23]^ However, complex preparation methods, low drug load efficiency, and poor long‐term stability of these nanomaterials inhibit their practical application. Thus, designing a stable chemotherapy/SDT synergistic nanoplatform is paramount.

Due to their excellent photostability, strong light‐harvest, and readily surface‐modifiable properties, polythiophenes have been widely used in biosensing, fluorescence imaging, and PDT of tumor.[^2^, ^24^, ^25^, ^26^, ^27^, ^28^, ^29^] Polythiophenes can behave as sonosensitizers because of their large π‐conjugated structure, controllable ROS generation, and easy molecular structure modification. Multiple functional groups can be modified on the polythiophene framework by adopting a copolymerization strategy, enabling it to have more functions. However, there are few reports on polythiophene derivatives for combining chemotherapy and SDT.^[30]^

**SCHEME 1:**
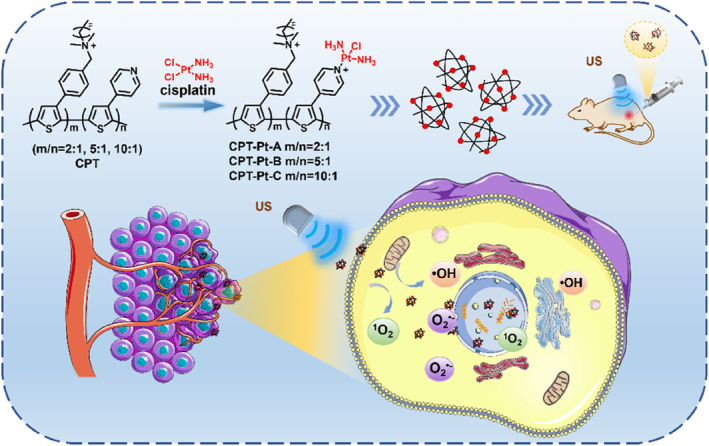
Synergistic chemotherapy and Sonodynamic therapy of tumor using **CPT‐Pts.**

The current study designed and synthesized a series of cisplatin‐coordinated copolythiophenes (**CPT‐Pts**) for the synergistic chemotherapy and SDT of tumors. As shown in Scheme 1, after copolymerizing 3‐phenylthiophene‐bearing quaternary ammonium and 4‐(3‐thienyl)pyridine, cisplatin was introduced through the coordination bond with pyridine to synthesize the polymeric sonosensitizers (**CPT‐Pts**). **CPT‐Pts** could produce ^1^O_2_, superoxide anion (O_2_^•‐^) and hydroxyl radical (•OH) under US irradiation, overcoming the SDT limitation in a hypoxic tumor environment. Moreover, introducing cisplatin allows the polymer to target the nucleus, and the toxic ROS produced in SDT can kill cancer cells more efficiently. Moreover, the sonodynamic toxicity and cytotoxicity of **CPT‐Pts** were precisely regulated by tuning the monomers ratio of the polythiophene. Herein, **CPT‐Pt‐B**, with a ratio of 5:1 of thiophene monomers containing quaternary ammonium salt and pyridine, was preferred as the combination therapy sonosensitizer for sonodynamic/chemotherapy synergistic therapy. This copolymerization strategy avoids the side effects of the high‐dose chemotherapy drug and makes up for the limitation of SDT depending on ultrasonic activation. The in vitro and in vivo treatment results indicated that **CPT‐Pt‐B** possesses a good tumor inhibition effect under US irradiation.

## 2 RESULTS AND DISCUSSION

**FIGURE 1:**
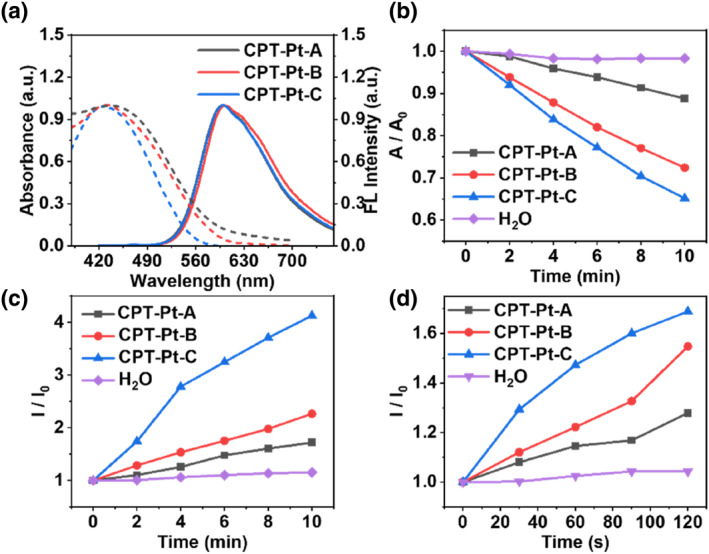
The optical properties and sonodynamic activation of **CPT‐Pts**. (a) Normalized absorption and fluorescence spectra of **CPT‐Pt‐A**, **CPT‐Pt‐B** and **CPT‐Pt‐C**. (b) The absorption decay rate of ABDA‐Na_2_, (c) The fluorescence intensity ratio of TA, and (d) DHR123 in the presence of **CPT‐Pts** under US irradiation (1.0 W·cm^−2^, 10 min or 120 s). TA, terephthalic acid; US, ultrasound.

The detailed synthesis and characterization of **CPT‐Pts** were presented in the (Figure S1–S4). The molecular weights of **CPT‐Pt‐A**, **CPT‐Pt‐B** and **CPT‐Pt‐C** were determined by Gel permeation chromatography (GPC), and the values were 6512, 6574 and 6783 g·mol^−1^, respectively (Figure S5). The Inductively Coupled Plasma Optical Emission Spectrometer (ICP‐OES) test result depicted 5.9, 2.5, and 1.9 μM cisplatin per 100 mg **CPT‐Pt‐A**, **CPT‐Pt‐B** and **CPT‐Pt‐C**, respectively. Due to the presence of hydrophilic quaternary ammonium, pyridine salts, and hydrophobic fatty long chains, **CPT‐Pts** could form nanoparticles via self‐assembly in aqueous solution. As shown in the Figure S6, the **CPT‐Pt‐B** disperses well in the aqueous solution and the average size of these nanoparticles was 95 ± 5 nm. Then, the ultrasonic stability of **CPT‐Pt‐B** was tested. As illustrated in Figure S7a, the potential of **CPT‐Pt‐B** did not change significantly before and after US. Besides, the absorption and fluorescence spectra did not change obviously before and after US (Figure S7b). This indicates that **CPT‐Pt‐B** has good ultrasonic stability. **CPT‐Pt‐A**, **CPT‐Pt‐B**, and **CPT‐Pt‐C** demonstrated one characterized peak at 437, 430, and 425 nm in the absorption spectrum (Figure 1a, dotted lines), with the maximal emission at around 600 nm (Figure 1a, full lines). The fluorescence quantum yield of **CPT‐Pts** with different monomer ratios (2:1, 5:1, 10:1) was determined to be 7.3%, 8.3%, and 9.2%, using sodium fluorescein as the reference. This is because the heavy atom effect increases the intersystem crossing probability, thus weakening the fluorescence.

9,10‐Anthracenediyl‐bis(methylene)dimalonic acid disodium (ABDA‐Na_2_), terephthalic acid (TA) and dihydrorhodamine 123 (DHR 123) helped evaluate the ^1^O_2_, •OH, and O_2_^•‐^ generation capabilities of **CPT‐Pts** in aqueous solution under US irradiation, respectively (Figure S8–S10). Using water as a blank control, with increasing monomer proportion of 3‐phenylthiophene‐bearing quaternary ammonium, the capability of **CPT‐Pts** generating ^1^O_2_, •OH and O_2_^•‐^ also increased (Figure 1b–d). These are due to the sonosensitive activity of **CPT‐Pts** is provided by the 3‐phenylthiophene bearing quaternary ammonium component, while **PPy‐Pt** does not possess sonosensitive activity (Figure S11). The above results indicated that the sonodynamic activity could be regulated by tuning the ratio of monomers. Regarding the content of cisplatin, we speculated that **CPT‐Pt‐B** had moderate cytotoxicity. Thus, **CPT‐Pt‐B** was selected for cell and tumor experiments.

**FIGURE 2:**
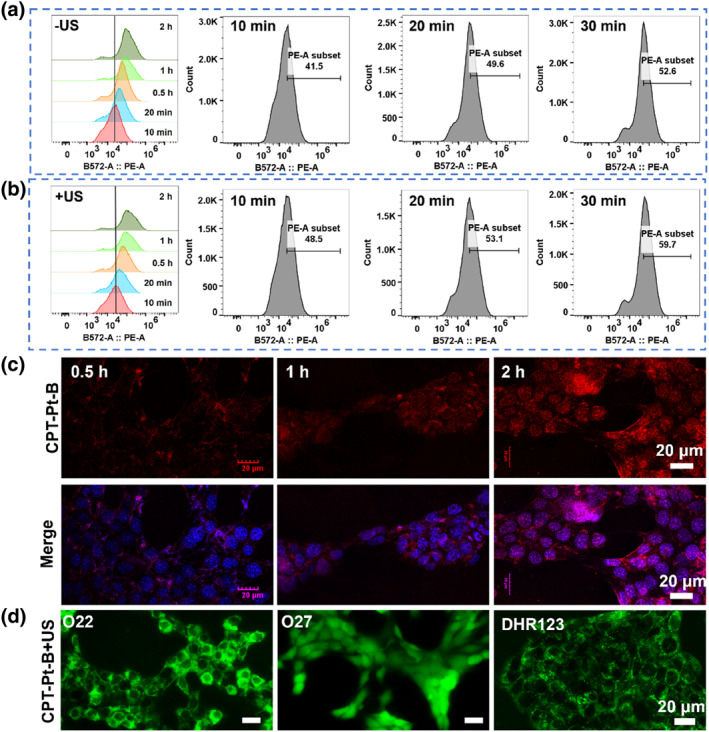
The cellular uptake and intracellular ROS generation. Flow cytometry statistical analysis of the cellular uptake assay (a) without or (b) with US (1.0 W/cm^−2^, 1 min). (c) Confocal microscopy observation of cellular fluorescence intensity of 4T1 cells incubated with DAPI and **CPT‐Pt‐B**. (d) Fluorescence images of ^1^O_2_, •OH and O_2_^•‐^ generation in 4T1 cells. US, ultrasound; ROS, reactive oxygen species.

Drugs can exert their anticancer activity after being endocytosed by cancer cells.^[31]^ The endocytosis of red fluorescent **CPT‐Pt‐B** by 4T1 cells was observed with a confocal laser scanning microscope (CLSM). Figure S12a,b shows that **CPT‐Pt‐B** uptake in 4T1 cells indicates a time‐dependent pattern. As time increased, the red fluorescence signal originated from **CPT‐Pt‐B** in the cells gradually increased, and US irradiation further accelerated the drug's entry into the cells. We further validated the cellular uptake assay using flow cytometry statistical analysis. As shown in Figure 2a,b, the intracellular fluorescence increased with incubation time. The cutoff fluorescence intensity as set from a histogram of control cells (used ‐US as the control group) was used to measure the percentage of **CPT‐Pt‐B**‐containing cells.^[32]^ For a short time (10, 20, and 30 min), the percentage of cells in the ‐US group with cutoff fluorescence intensity was lower than in the +US group at the same incubation time, which proved that US irradiation can promote the cellular uptake of drugs to a certain extent. Moreover, introducing cisplatin helps the sonosensitizer to enter the nucleus. The nuclear dye 4,6‐Diamidino‐2‐phenylindole dihydrochloride (DAPI) helped confirm the above conclusions (Figure 2c).

The fluorescent inverted microscope helped directly observe the intracellular ROS production by **CPT‐Pt‐B** under US irradiation while using O22, O27 and DHR123 as the ^1^O_2_, •OH and O_2_^•‐^ probes, respectively. Figure S12c shows no green fluorescence in the phosphate buffer saline (PBS), PBS + US, and **CPT‐Pt‐B** groups. This indicated that ^1^O_2_, •OH or O_2_^•‐^ was not produced by US or **CPT‐Pt‐B** alone. On the contrary, strong green fluorescence was observed in the **CPT‐Pt‐B** + US group, indicating that **CPT‐Pt‐B** could significantly generate ^1^O_2_, •OH and O_2_^•‐^ in cells under US irradiation (Figure 2d).

**FIGURE 3:**
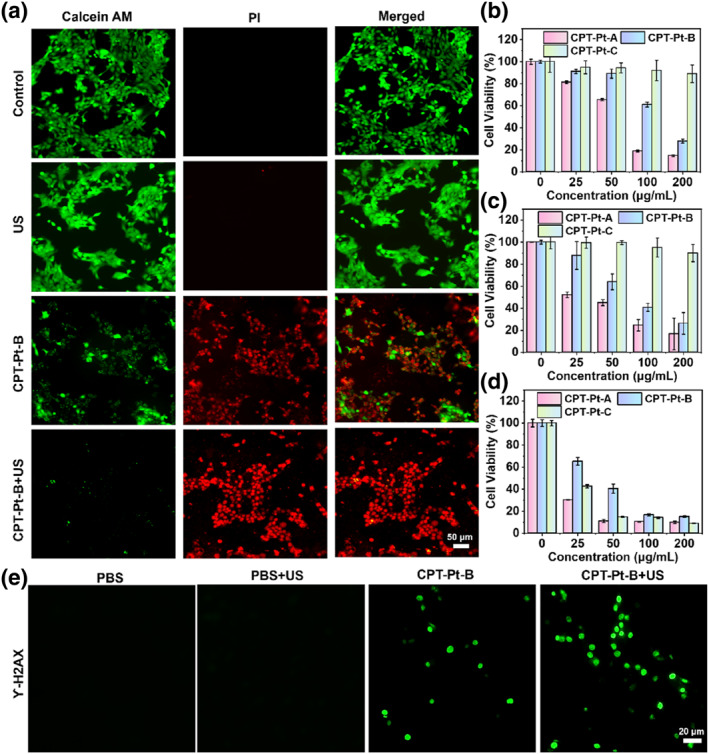
In vitro anticancer activity of **CPT‐Pt‐B**. (a) The fluorescence images of 4T1 cells were stained using Calcein‐AM (green, viable) and PI (red, dead) after different treatments. Scale bar: 50 μm. The viability of 4T1 cells was incubated using different concentrations of **CPT‐Pts** for (b) 24 h and (c) 48 h without US irradiation. (d) The viability of 4T1 cells was incubated using different concentrations of **CPT‐Pts** with US irradiation (1.0 W·cm^−2^, 1 min). (e) The fluorescence images of DNA damage in 4T1 cells post‐different treatments. AM, acetoxymethyl easter; PI, propidiumiodide; US, ultrasound.

According to cell uptake efficiency and significant ROS production ability of **CPT‐Pt‐B**, the synergistic chemotherapy effect and SDT on 4T1 cells was assessed using the Cell Viability/Cytotoxicity Assay Kit comprising calcium protein acetoxymethyl easter and propidiumiodide (PI). Figure 3a depicts no visible red fluorescence in the PBS and US alone groups. This was consistent with the previously mentioned results, where the control and US groups could not generate ROS. However, a particular cell death existed by incubating with the **CPT‐Pt‐B** even without US irradiation, because of the chemotherapy effect of cisplatin. Moreover, the **CPT‐Pt‐B** + US group showed the most pronounced cell death. These results indicated that combining SDT and chemotherapy can effectively kill cancer cells.

Furthermore, the 3‐(4,5‐Dimethylthiazol‐2‐yl)‐2,5‐diphenyltetrazolium bromide (MTT) assay helped quantitatively assess the viability of 4T1 cells after different treatments. Without US irradiation, the cell viability indicated apparent concentration and incubation time dependence. As depicted in Figure 3b, 85.3% cells were killed in the presence of 200 μg·ml^−1^ of **CPT‐Pt‐A** for 24 h. While only 11.0% cells were killed by the **CPT‐Pt‐C**. The IC_50_ value of **CPT‐Pt‐A**, **CPT‐Pt‐B** and **CPT‐Pt‐C** were 71.8, 222.1 and 1528 μg·ml^−1^, respectively, which are smaller than that of cisplatin (4351 μg·mL^−1^). This avoids the problem of high dose of chemotherapy drugs. Consistent results were also obtained by incubation for 48h. **CPT‐Pt‐A** killed nearly 50% of the cells after 48 h of incubation, while the same concentration of **CPT‐Pt‐B** only killed 12.2%. However, treatment is a long‐term process, and **CPT‐Pt‐A** is too cytotoxic to be suitable for subsequent therapy considering the manifestation of synergistic therapeutic effects. Besides, 50 μg·mL^−1^ of **CPT‐Pt‐B** killed 36.2% of the cells after 48 h. After US irradiation, **CPT‐Pts** was found to be effective in killing cells (Figure 3d). Moderate cytotoxicity combined with SDT of **CPT‐Pt‐B** maybe the ideal agent for cancer therapy due to excellent therapeutic effect and the biological safety.

After entering the cancer cells, cisplatin induces DNA damage, leading to cell cycle arrest and inducing cell death. To determine whether **CPT‐Pt‐B** is capable of therapeutic intervention through this mechanism, the immunofluorescence staining assay (green) of *γ*‐H2AX (DNA damage marker) was performed. As shown in Figure 3e, strong green fluorescence signal in the nucleus of 4T1 cells treated using **CPT‐Pt‐B** and **CPT‐Pt‐B** + US were observed. However, no fluorescence signals could be observed in the PBS or PBS + US group cells.

**FIGURE 4:**
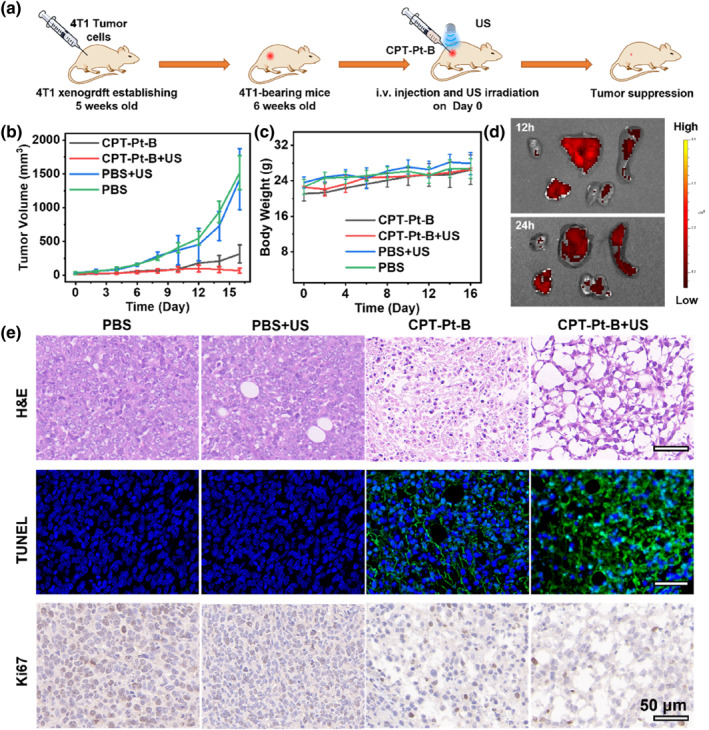
In vivo anti‐cancer activity of **CPT‐Pt‐B**. (a) Schematic illustration of the treatment schedule. (b) Tumor growth inhibition curves after various treatments. (c) Changes in body weight of mice after multiple treatments. (d) Ex‐vivo imaging of major organs after 12 and 24 h of administering **CPT‐Pt‐B.** (e) H&E, TUNEL, and Ki67 staining of the tumor tissue slices after different treatments. (Scale bars: 50 μm).

The anticancer capability of **CPT‐Pt‐B** was studied within the 4T1 tumor‐bearing mouse model (Figure 4a). We injected 4T1 cells inside the right hind limb of Balb/c mice to construct tumor model mice, and randomly divided them into four groups (*n* = 5): (1) PBS, (2) PBS + US, (3) **CPT‐Pt‐B**, (4) **CPT‐Pt‐B** + US. PBS or **CPT‐Pt‐B** aqueous solution was intratumor injected into the mice. As depicted in Figure 4b, for mice in control groups (1) and (2), the tumor volume increased significantly. This proved that PBS or US treatment showed no inhibitory effect on the tumor. In groups (3) and (4), the tumor volume was significantly restricted while treated with **CPT‐Pt‐B**. However, group (3) of mice showed tumor recurrence after the 10th day, while group (4) did not. These results depict that **CPT‐Pt‐B** could effectively inhibit tumor growth via chemotherapy combined with SDT. Moreover, the mice showed no significant weight loss throughout the treatment (Figure 4c), establishing the benign biosafety of **CPT‐Pt‐B**.

Next, we injected **CPT‐Pt‐B** into mice through the tail vein and observed fluorescence imaging of major organs to investigate the metabolism of **CPT‐Pt‐B** in vivo. According to Figure 4d, after 12 h, the fluorescence in the liver was relatively strong, which decreased after 24 h. The results showed that the liver mainly metabolized **CPT‐Pt‐B**. After 16 days of treatment, the animal models were sacrificed to validate the anti‐tumor effect. Tumorous tissues excised from the four groups were studied using the histological analysis after hematoxylin and eosin (H&E) staining, terminal deoxynucleotidyl transferase‐mediated dUTP‐biotin nick end labeling (TUNEL), and Ki67 immunofluorescence staining helped measure the tumor proliferation capacity (Figure 4e). H&E staining showed more significant nuclear fragmentation and severe necrosis of tumor cells in mice treated with **CPT‐Pt‐B** and **CPT‐Pt‐B** + US than in other groups. The TUNEL staining results were in agreement with H&E staining. No green fluorescence could be observed in the PBS and US alone group, indicating that PBS and US would not induce tumor apoptosis. However, different degrees of green fluorescence were observed in the **CPT‐Pt‐B** and **CPT‐Pt‐B** + US groups, indicating different degrees of apoptosis. Similarly, the results of the Ki67 sections can also support the above conclusions. The nucleus contracted, and the cytoplasmic space enlarged, suggesting the inhibitory effect of **CPT‐Pt‐B** + US on the proliferation of tumor cells was significantly higher than in other groups. **CPT‐Pt‐B** biosafety was evaluated by removing major organs for histopathological analysis after the 16‐day treatment cycle (Figure S13a). Compared with the PBS group, the results indicated no visible injury in the main organs (heart, liver, spleen, lung, and kidney) of mice in the **CPT‐Pt‐B** + US group, establishing the biosafety of **CPT‐Pt‐B**. Besides, the **CPT‐Pt‐B** and PBS were injected into the mice through the tail vein, and after 24 h later, blood samples were collected for routine blood test. As depicted in Figure S13b, after treatment with **CPT‐Pt‐B**, there were no significant differences in all the indexes compared with PBS group, which indicated that the drug had good biological safety.

## 3 CONCLUSION

Therefore, we designed and synthesized a series of cisplatin‐coordinated copolythiophene (**CPT‐Pts**) for the synergistic chemotherapy and SDT of tumors. We regulated its sonodynamic toxicity and cytotoxicity by controlling the ratio of monomers. Moreover, we explored the most efficient polythiophene derivative for sonodynamic/chemotherapy synergistic therapy. The in vitro and in vivo treatment results revealed that **CPT‐Pt‐B** showed a good tumor inhibition effect under US irradiation. This copolymerization strategy avoids the side effects of the high‐dose chemotherapy drug while covering the limitation of SDT depending on ultrasonic activation, effectively inhibiting cancer cells and tumors.

## 4 EXPERIMENTAL SECTION/METHODS

*Materials*: ABDA was procured from Shanghai Aladdin Biochemical Technology Co., Ltd. TA was provided by Shanghai Macklin Biochemical Technology Co., Ltd. O22, O27, and DHR123 probe were purchased from Beijing BioLab. Technology Co., Ltd. MTT was obtained from Sigma‐Aldrich Trading. Calcein‐AM and PI were procured from Heowns Biochem Technologies, LLC, Tianjin. Co., Ltd. DNA Damage Assay Kit was bought from Beijing Beyotime Biotechnology Co., Ltd.

*Instruments*: The UV‐vis‐NIR absorption and fluorescence spectra were evaluated on Shimadzu UV2600 and RF6000 spectrophotometers, respectively. PerkinElmer Avio 500 ICP‐OES helped to determine the cisplatin contents. The molecular weights were determined by Agilent 1260 infinity II GPC. Fluorescence cellular images were obtained under an inverted fluorescence microscope (Leica DMIL LED) and a CLSM (Leica SP8). Flow cytometry was done through a BD Accuri™ C6 Plus (BD Biosciences) system. MTT cell viability experiments were performed using an enzyme‐labeled instrument (Varioskan LUX).

*Synthesis of* ***CPT‐Pts***: Compounds 1, 2, and 3 were synthesized based on our previous reports.[^33^, ^34^] All the polymers in this paper were prepared using oxidative polymerization with FeCl_3_ under nitrogen. The specific synthesis steps are: 30 mL of dry CHCl_3_ was added under nitrogen to a degassed mixture of four equivalents of FeCl_3_ and one equivalent of the corresponding monomers. The reaction mixture was stirred at room temperature for 48 h. The product was washed using methanol, and the precipitate was obtained. Finally, a dark red solid was obtained by drying under a vacuum. For copolymerization, monomers **2** and **3** with different mole ratios (2:1, 5:1, 10:1) were used to provide the corresponding copolythiophenes. (CPT‐A, CPT‐B and CPT‐C, respectively). The polymerization of monomer 3 gives the corresponding homopolythiophenes **PPy**. Cisplatin and equivalent AgNO_3_ were dissolved in N,N‐Dimethylformamide (DMF), and then reacted at 55°C for 24 h. The AgCl precipitate was removed by centrifugation and the supernatant was taken, which was a light‐yellow liquid. The liquid was mixed with the same monomer equivalent of **CPT** or **PPy**, and reacted at 55°C for the next 24 h in DMF. The dark red solution was diluted with water, and dialyzed for 48 h to remove DMF, and lyophilized to obtain dark red powder for **CPT‐Pt‐A, CPT‐Pt‐B**, **CPT‐Pt‐C** and **PPy‐Pt** respectively.

*Determination of* ^*1*^ *O*_*2,*_ *•OH and O*_*2*_^*•‐*^ *in aqueous solution*: ABDA‐Na_2_ helped detect ^1^O_2_ generated by **CPT‐Pts** under US irradiation. ABDA‐Na_2_ (50 μL) was added into **CPT‐Pts** aqueous solution (2 mL, 25 μM), and the mixture was irradiated with US through an ultrasonic therapy instrument (1.0 MHz, 1.0 W·cm^−2^) for five cycles, each for 2 min. The UV‐vis‐NIR absorption spectra of ^1^O_2_ captured by ADBA were measured. TA helped assay the •OH generation capability of **CPT‐Pts**. The assay was conducted by adding 100 μL of TA (50 mM) to 2 mL of **CPT‐Pts** (25 μM). The fluorescence spectra of the mixture were scanned every 2 min under US irradiation (*λ*_ex_ = 320 nm). DHR123 was used to detect the O_2_^•‐^ generated by **CPT‐Pts** (25 μM). The assay was conducted by adding 1 μL of TA (1 mg·mL^−1^) to 2 mL of **CPT‐Pts** (25 μM). The fluorescence spectra of the mixture were scanned every 30 s under US irradiation (*λ*_ex_ = 504 nm).

*Cell uptake assay*: The 4T1 cells were incubated with **CPT‐Pt‐B** for different times (0, 0.5, 1, 2, 4, 8h). After drug addition, US was applied immediately in the US group (1.0 MHz, 1.0 W·cm^−2^). Then, all the cells were washed three times with PBS. Finally, the cells across all groups were imaged with an inverted fluorescence microscope.

*Flow cytometry*: The 4T1 cells were incubated with **CPT‐Pt‐B** for different times (0, 10 min, 20 min, 0.5, 1, 2 h). After drug addition, US was applied immediately in the US group (1.0 MHz, 1.0 W·cm^−2^). Then, all the cells were washed three times with PBS. Cells were collected by digestion with trypsin in a centrifuge tube and centrifuged. After resuspension in PBS, the samples were analyzed by flow cytometry.

*Determination of intracellullar* ^1^O_2,_ •OH and O_2_^•^: The 4T1 cells were divided into four groups (PBS, PBS + US, **CPT‐Pts**, and **CPT‐Pts** + US). First, the cells in each group were incubated in a 37°C cell incubator for 24 h. Subsequently, the medium culturing cells in PBS and US groups were replaced with a new 1640 culture medium. On the contrary, the medium used to culture cells in the **CPT‐Pts** and **CPT‐Pts** + US groups was replaced using a 1640 culture medium containing **CPT‐Pts**. After incubation for 4 h, O22, O27, and DHR123 probes were added and continued for another 30 min. After that, all the cells were washed thrice with PBS, and the cells in the US and **CPT‐Pts** + US groups were treated with an ultrasonic therapeutic apparatus (1.0 MHz, 1.0 W·cm^−2^). Finally, the cells across all groups were imaged with an inverted fluorescence microscope.

*MTT cell experiment*: The 4T1 cells were evenly planted across 96‐well plates and cultured at 37°C for 24 h inside a cell incubator with 5% CO_2_. The 1640 culture group containing different **CPT‐Pts** concentrations was replaced for incubation. The US group cells were incubated for 4 h, and an ultrasonic therapy instrument was utilized for US (1.0 MHz, 1.0 W·cm^−2^, 1 min). After 24 h, the medium was discarded, and 200 μL cell medium containing MTT (10%) was added to each well. After 4 h, the medium was discarded, and 200 μL dimethyl sulfoxide (DMSO) was added to each well. After 10 min, DMSO was tested using an enzyme‐labeled instrument.

*AM/PI staining*: The 4T1 cells were divided into four groups, that is, PBS, PBS + US, **CPT‐Pt‐B**, **CPT‐Pt‐B** + US groups, and evenly grown using 24‐well plates. After incubating for 24 h in the cell incubator, the **CPT‐Pt‐B** and **CPT‐Pt‐B** + US groups were replaced using fresh 1640 medium containing **CPT‐Pt‐B** (50 μg·mL^−1^). Moreover, the PBS and PBS + US groups were replaced using a mixture of PBS and 1640 medium and incubated for 4 h. The cells in each group were stained with calcein‐AM (2 μM, 10 μL)‐PI solution (2 μM, 10 μL) for 10 min (the depicts calcein‐AM shows green fluorescence for live cells; PI stains for dead cells, indicating red fluorescence). Subsequently, US treatment (1.0 MHz, 1.0 W·cm^−2^, 1 min) was provided to the US and **CPT‐Pt‐B** + US groups using an ultrasonic therapy instrument. After washing thrice with PBS, the cellular staining in each group was observed using a fluorescent inverted microscope.

*DNA damage test*: The 4T1 cells were divided into four groups, that is, PBS, PBS + US, **CPT‐Pt‐B**, **CPT‐Pt‐B** + US groups, and evenly grown across 24‐well plates. After incubating for 24 h in the cell incubator, the **CPT‐Pt‐B** and **CPT‐Pt‐B** + US groups were replaced using fresh 1640 medium containing **CPT‐Pt‐B** (50 μg·mL^−1^). Then, the PBS and PBS + US groups were replaced using a mixture of PBS and 1640 medium and incubated for 4 h. Subsequently, US treatment (1.0 MHz, 1.0 W·cm^−2^, 1 min) was performed on the US and **CPT‐Pt‐B** + US groups using an ultrasonic therapy instrument. Then, cells in each group were treated with a DNA Damage Assay Kit. After washing the cells three times with PBS, their staining in each group was observed using a fluorescent inverted microscope.

*Animal model*: Animal experiments were performed under the approval of the Hunan Normal University Ethics Committee (No. D2022024). Balb/c male mice (8 weeks) were purchased from Hunan SJA Laboratory Animal Co., Ltd. We evenly grew eight boxes of 4T1 cells in a 90 mm^2^ culture dish to establish a mouse tumor model. When the cells increased to above 80%, the cells were digested and centrifuged. Then, 2 mL of culture medium was added and blown well using a pipette, and the 4T1 cells were injected under the back skin of two mice (1 mL of each). After about 2 weeks, the tumor was peeled and divided into uniform small spieces when the tumor grew to 800 mm^3^ and transplanted to the subcutaneous dorsal surface of healthy mice. After about 7 days, the tumors grew to 100 mm^3^, and the mouse experiment was started. The mice were randomly divided into four groups, and the **CPT‐Pt‐B** and **CPT‐Pt‐B** + US groups were injected intratumorally using the **CPT‐Pt‐B** solution (50 μg·mL^−1^, 200 μL). After waiting for 10 min, the coupling agent was spread on the US probe, and US treatment (1.0 MHz, 1.0 W·cm^−2^ for 5 min) was applied to the tumors of mice. Tumor growth and weight changes in mice were recorded over 14 days.

*H&E, Ki67, and TUNEL section experiments*: Four 4T1 tumor‐bearing mice were treated with PBS, PBS + US **CPT‐Pt‐B**, **CPT‐Pt‐B** + US (200 μL, 50 μg·mL^−1^ of **CPT‐Pt‐B** or 200 μL of PBS). Then, the tumor tissues were dissected and fixed using 4% paraformaldehyde. Next, H&E, Ki67, and TUNEL stained sections under the aegis of Wuhan Sevier Biotechnology Co. Ltd.

*Routine blood tests*: The drugs and PBS were injected into the healthy mice through the tail vein, and after 24 h later, blood samples were collected for routine blood test.

## CONFLICT OF INTEREST STATEMENT

The authors declare no conflicts of interests.

## ETHICS STATEMENT

Animal experiments were performed under the approval of the Hunan Normal University Ethics Committee (No. D2022024).

## Supporting information

Supporting Information S1

## Data Availability

The data that support the findings of this study are available in the supplementary material of this article.
