# Supplementary material for: Cisplatin‐coordinated copolythiophene for synergistic chemotherapy and sonodynamic therapy of tumor
Source: Smart Mol. 2024 Jun 7;2(3):e20240003. doi: 10.1002/smo.20240003 (PMC12118247; doi:10.1002/smo.20240003)
Supplement: Supplementary file 1 — Supporting Information S1 [file SMO2-2-e20240003-s001.docx]

Supporting Information

Cisplatin-Coordinated Copolythiophene for Synergistic Chemotherapy and Sonodynamic Therapy of tumor

Yuanyu Tang, E Pang, Pan Zhu, Qiuxia Tan, Shaojing Zhao,* Benhua Wang, Chaoyi Yao, Xiangzhi Song, and Minhuan Lan*

Hunan Provincial Key Laboratory of Micro & Nano Materials Interface Science, College of Chemistry and Chemical Engineering, Central South University, Changsha, Hunan, 410083, P. R. China.

E-mails: Dr. S. Zhao, 31180030@csu.edu.cn; Prof. M. Lan, minhuanlan@csu.edu.cn


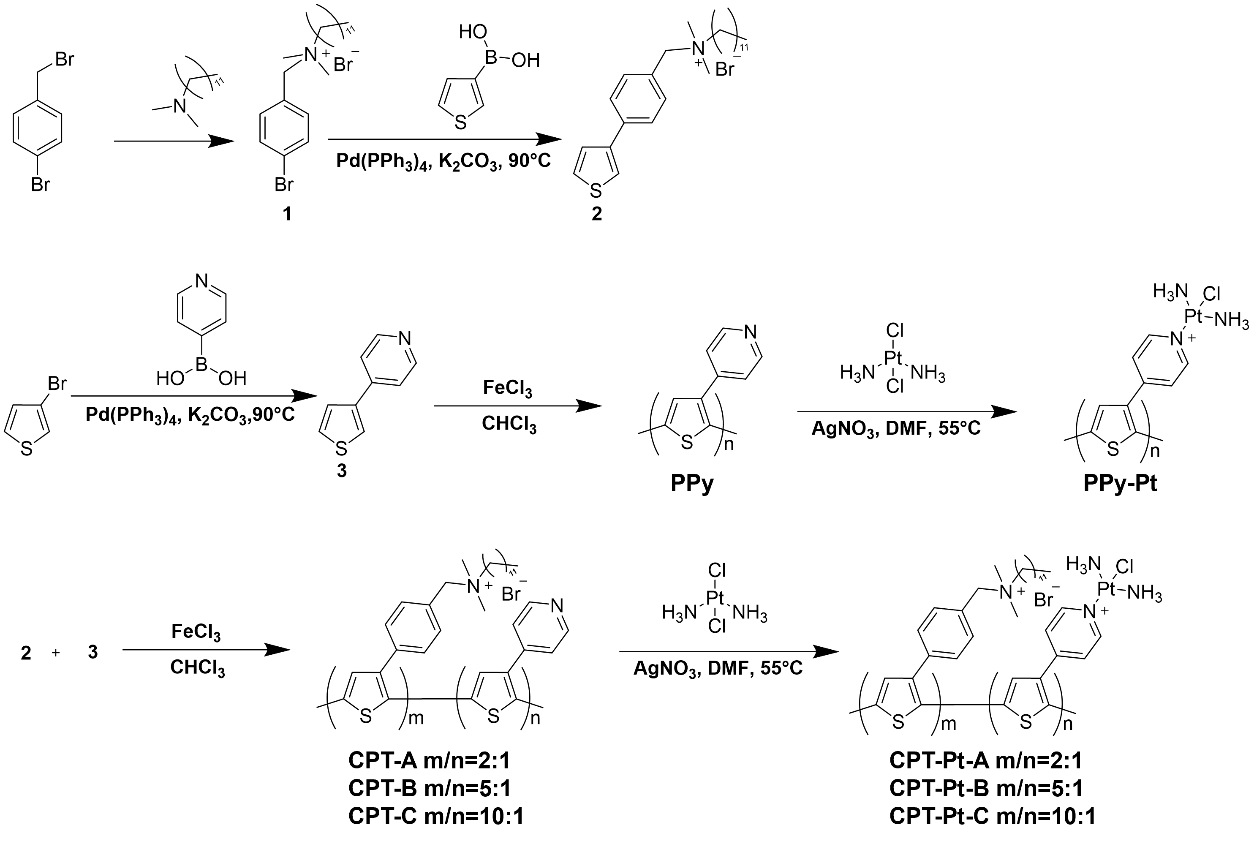


**Figure S1.** Synthetic routes of **CPT-Pts**.


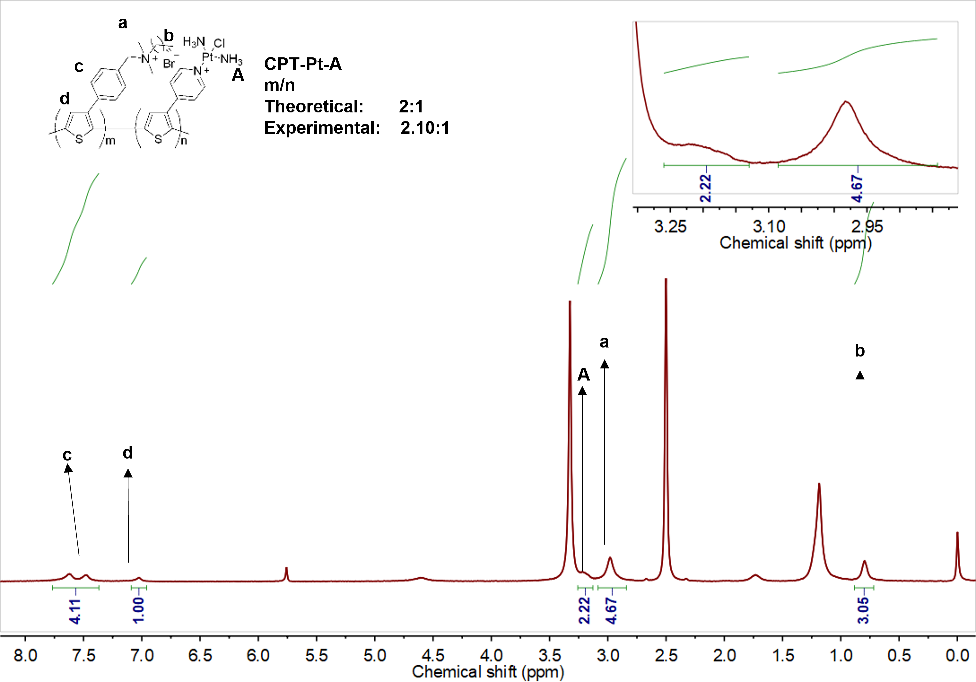


**Figure S2.** ^1^H NMR spectra of **CPT-Pt-A** (400 MHz, DMSO, TMS, ppm).


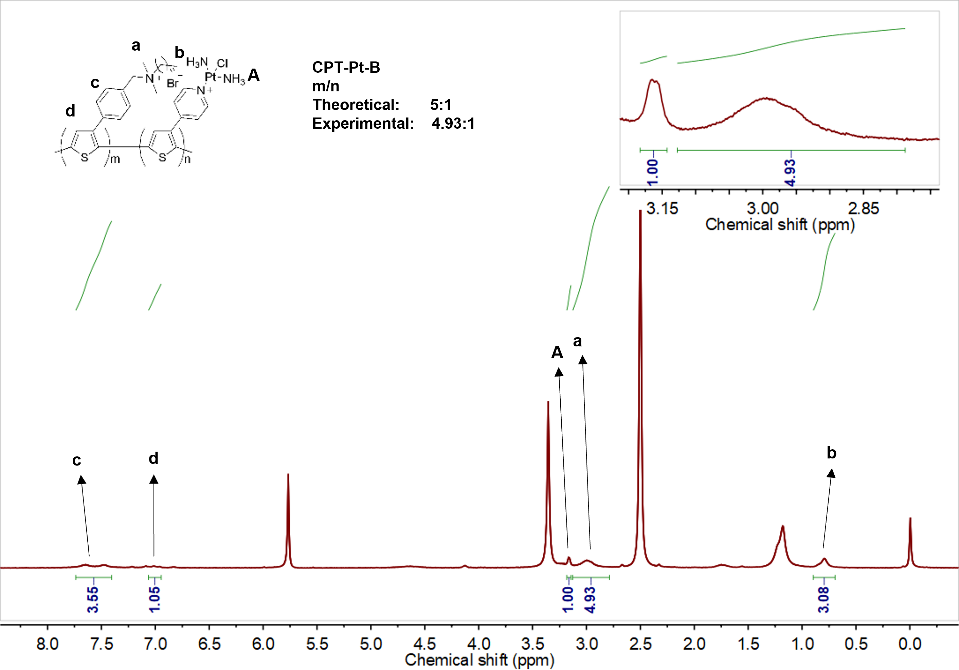


**Figure S3.** ^1^H NMR spectra of **CPT-Pt-B** (400 MHz, DMSO, TMS, ppm).


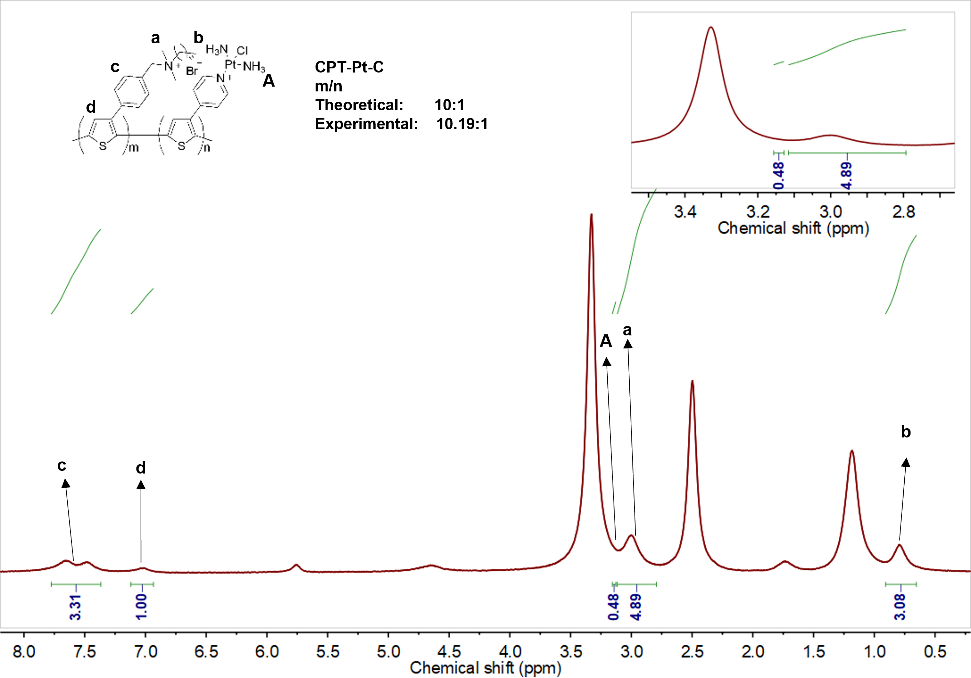


**Figure S4.** ^1^H NMR spectra of **CPT-Pt-C** (400 MHz, DMSO, TMS, ppm).


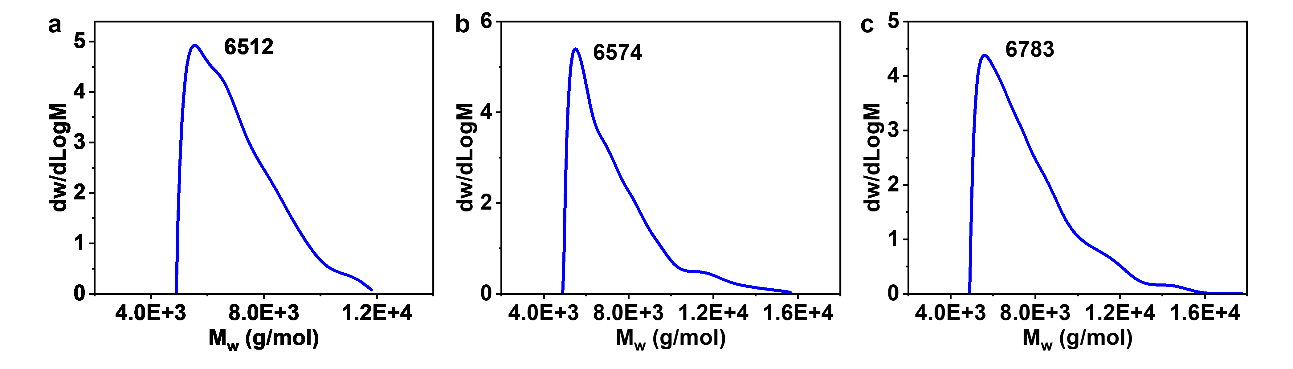


**Figure S5.** Molecular weight profile of (a) **CPT-Pt-A,** (b) **CPT-Pt-B** and (c) **CPT-Pt-C**.

**
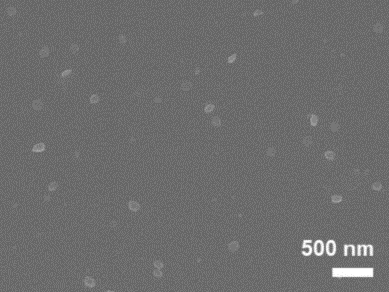
**

**Figure S6.** SEM image of **CPT-Pt-B**.


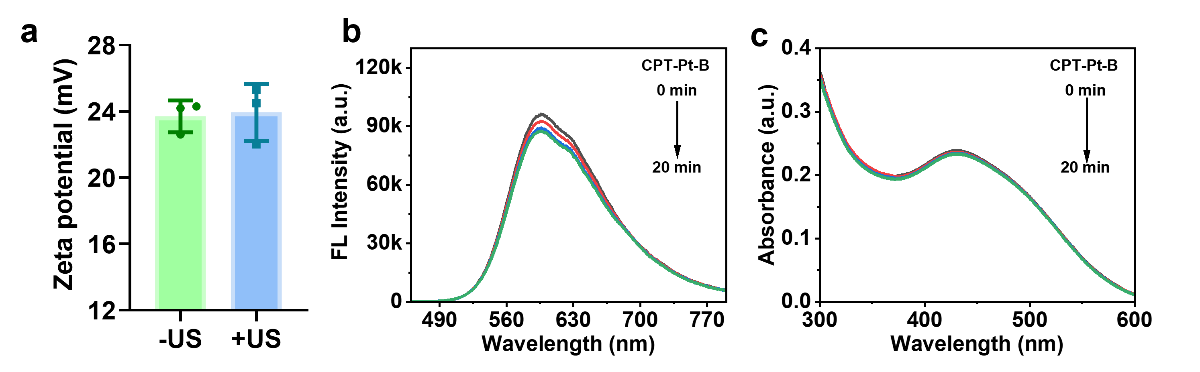


**Figure S7.** (a) Zeta potential of **CPT-Pt-B** before and after ultrasound for 20 min. Time-dependent absorption (b) and fluorescence (c) spectra of **CPT-Pt-B** after ultrasound irradiation.


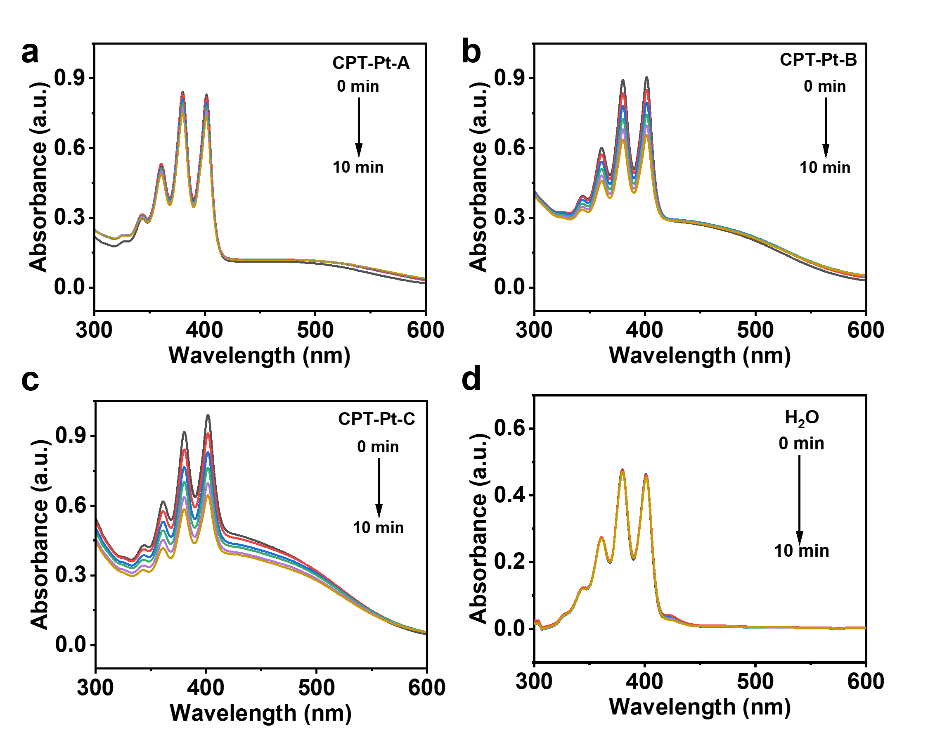


**Figure S8.** Absorption spectra of ABDA in the presence of (a-c) **CPT-Pts** and (d) H_2_O under US irradiation (1.0 W·cm^-2^, 10 min).


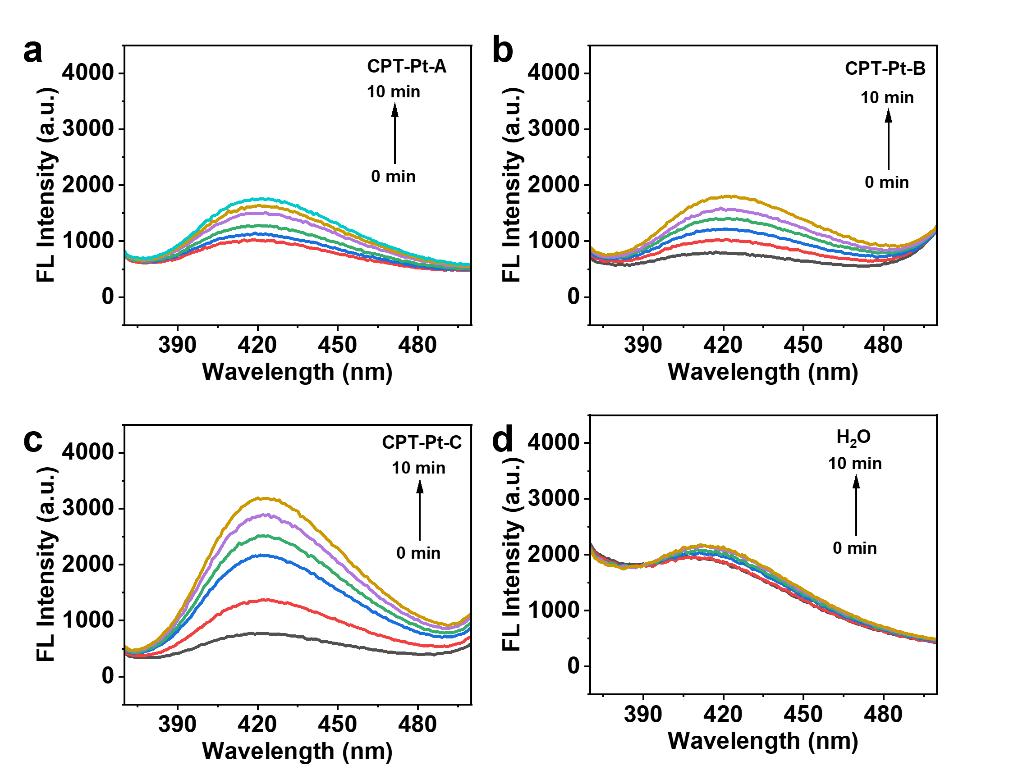


**Figure S9.** Fluorescence spectra of TA in the presence of (a-c) **CPT-Pts** and (d) H_2_O under US irradiation (1.0 W·cm^-2^, 10 min).


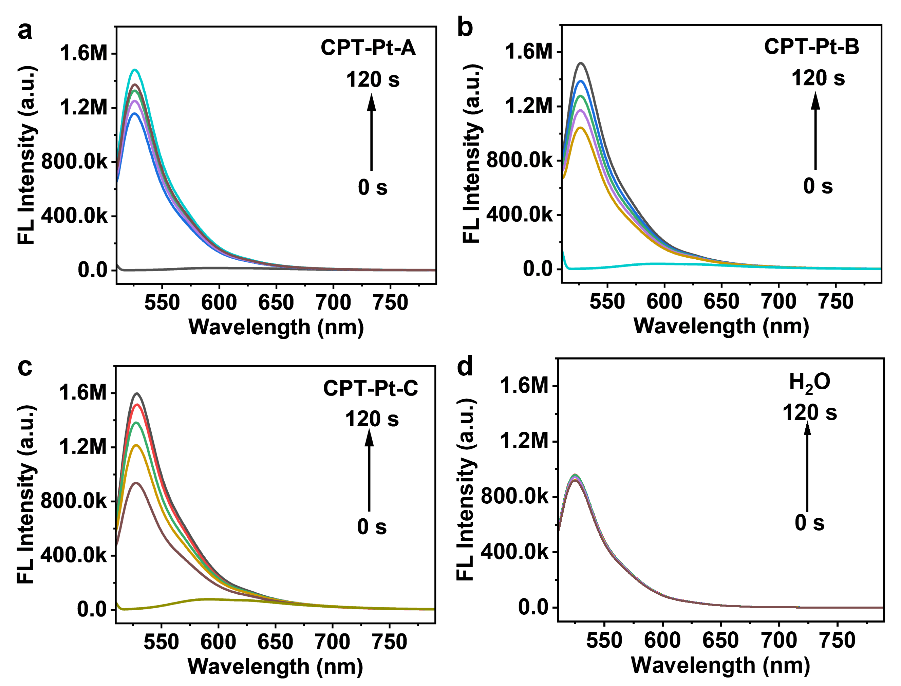


**Figure S10.** Fluorescence spectra of DHR123 in the presence of (a-c) **CPT-Pts** and (d) H_2_O under US irradiation (1.0 W·cm^-2^, 120 s).


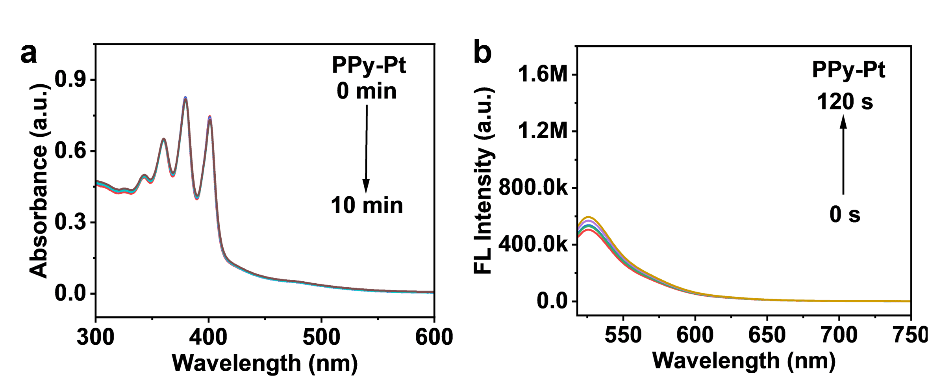


**Figure S11.** (a) Absorption spectra of ABDA in the presence of **PPy-Pt** under US irradiation (1.0 W·cm^-2^, 10 min). (b) Fluorescence spectra of DHR123 in the presence of under US irradiation (1.0 W·cm^-2^, 120 s).


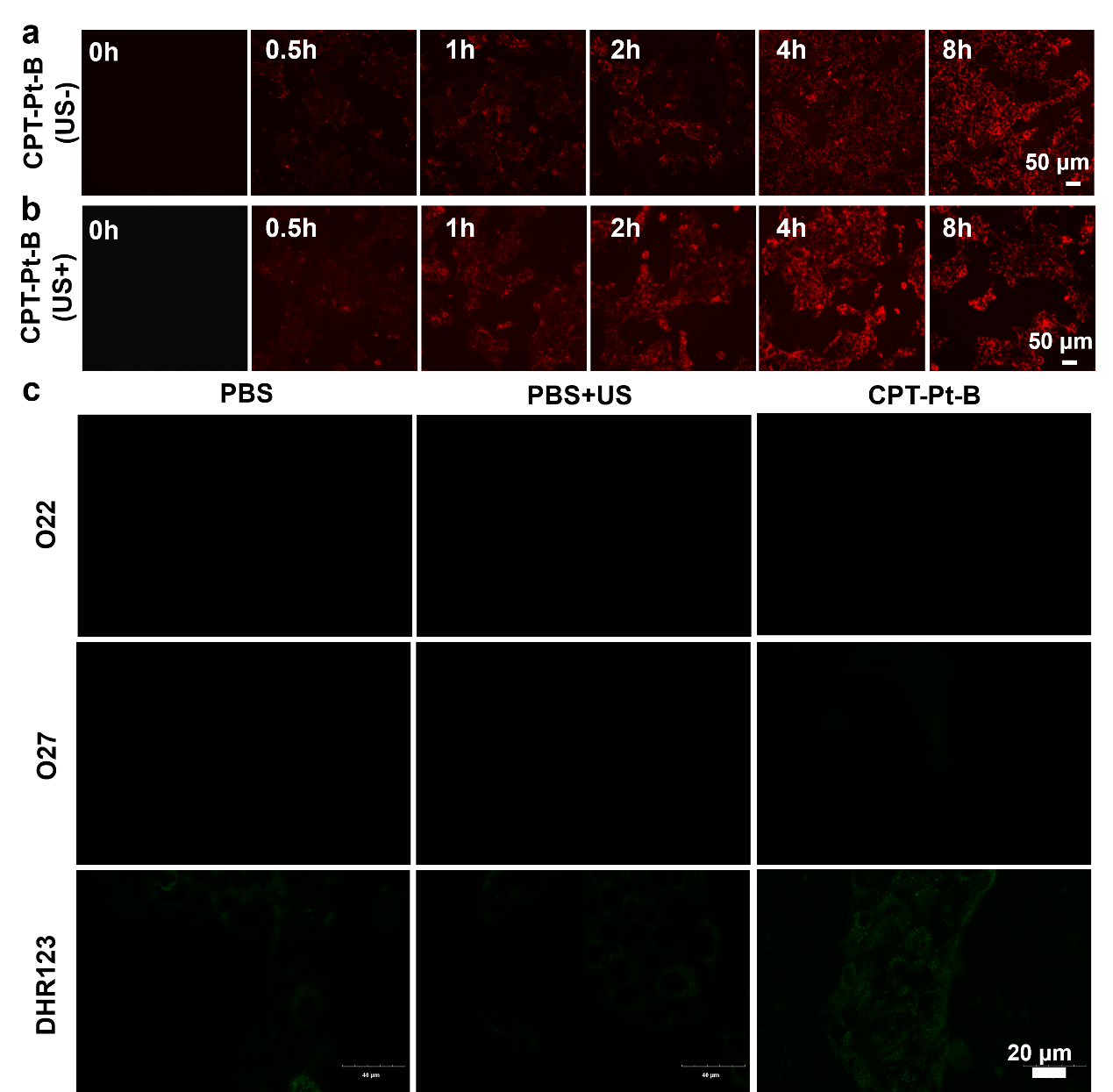


**Figure S12.** The cellular uptake and intracellular ROS generation. The fluorescence images of 4T1 cells co-incubated using **CPT-Pt-B** for 0.5 h, 1 h, 2 h, 4 h, and 8 h without (a) or with (b) US irradiation (1.0 W·cm^-2^, 1 min). The fluorescence images of (c) ^1^O_2_, (d) •OH and O_2_^•-^ generation of 4T1 cells incubated with PBS and **CPT-Pt-B** alone.

**
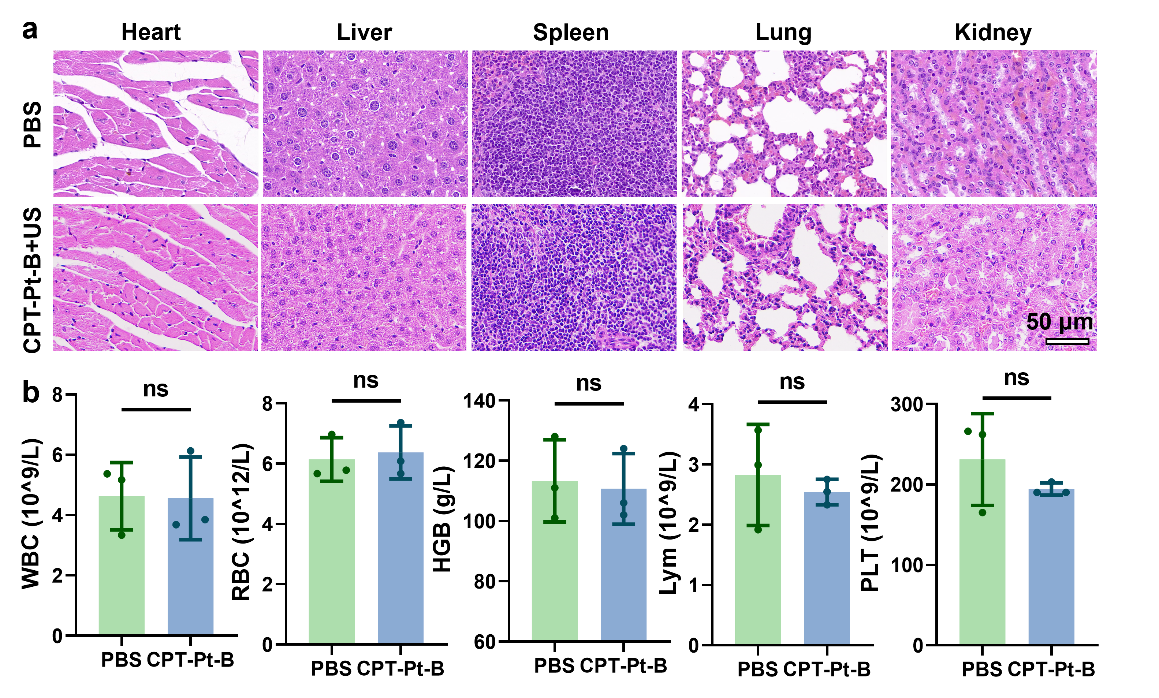
**

**Figure S13.** (a) H&E staining of the major organ sections after 16 days different treatments. (b) Results of routine blood test after PBS and **CPT-Pt-B** treatment.
